# Molecular mechanisms in the pathogenesis of *N*-nitrosodimethylamine induced hepatic fibrosis

**DOI:** 10.1038/s41419-018-1272-8

**Published:** 2019-01-08

**Authors:** Joseph George, Mutsumi Tsuchishima, Mikihiro Tsutsumi

**Affiliations:** 1 Division of Liver Diseases, Department of Medicine, Icahn School of Medicine at Mount Sinai, 1425 Madison Avenue, New York, NY 10029 USA; 2 Department of Hepatology, Kanazawa Medical University, Uchinada, Ishikawa 920-0293 Japan

## Abstract

Hepatic fibrosis is marked by excessive synthesis and deposition of connective tissue proteins, especially interstitial collagens in the extracellular matrix of the liver. It is a result of an abnormal wound healing in response to chronic liver injury from various causes such as ethanol, viruses, toxins, drugs, or cholestasis. The chronic stimuli involved in the initiation of fibrosis leads to oxidative stress and generation of reactive oxygen species that serve as mediators of molecular events involved in the pathogenesis of hepatic fibrosis. These processes lead to cellular injury and initiate inflammatory responses releasing a variety of cytokines and growth factors that trigger activation and transformation of resting hepatic stellate cells into myofibroblast like cells, which in turn start excessive synthesis of connective tissue proteins, especially collagens. Uncontrolled and extensive fibrosis results in distortion of lobular architecture of the liver leading to nodular formation and cirrhosis. The perpetual injury and regeneration process could also results in genomic aberrations and mutations that lead to the development of hepatocellular carcinoma. This review covers most aspects of the molecular mechanisms involved in the pathogenesis of hepatic fibrosis with special emphasize on *N*-Nitrosodimethylamine (NDMA; Dimethylnitorsmaine, DMN) as the inducing agent.

## Facts

- Hepatic fibrosis is due to the excessive synthesis and deposition of connective tissue proteins, especially interstitial collagens in the extracellular matrix of the liver.
- Abnormal wound healing in response to chronic liver injury is responsible for the pathogenesis of hepatic fibrosis.
- The key event involved in the pathogenesis of hepatic fibrosis is the activation and transformation of resting hepatic stellate cells into myofibroblast like cells and subsequent upregulation of hundreds of genes.
- Oxidative stress and generation of reactive oxygen species (ROS) serve as mediators of the molecular events implicated in hepatic fibrosis.
- The precise molecular mechanism involved in the pathogenesis and progression of hepatic fibrosis is not clear.

## Open Questions

- Exploration of methods to arrest activation and transformation of hepatic stellate cells into myofibroblast like cells.
- Development of potent antioxidant therapy that could destroy reactive oxygen species, which serve as mediators for the pathogenesis of hepatic fibrosis.
- Identification of appropriate gene targets that block the pathogenesis of hepatic fibrosis.
- Development of methods to reverse the process of hepatic fibrosis.
- Identification of strategies to prevent transformation of hepatic fibrosis to liver cirrhosis.

## Introduction

Hepatic fibrosis and liver cirrhosis are chronic diseases and serious health problems worldwide. Excessive synthesis and deposition of connective tissue proteins, especially interstitial collagens in the extracellular matrix of the liver is the hallmark of hepatic fibrosis^1–6^. It is a dynamic process resulting from a continuous wound healing response to a variety of chronic stimuli, such as ethanol, viruses, toxins, drugs, or cholestasis. The process of hepatic fibrosis is initiated with cellular oxidative stress and production of reactive oxygen species (ROS) that serve as mediators of molecular events involved in the pathogenesis of hepatic fibrosis^7–10^. These processes results in cellular injury and release a variety of cytokines and growth factors that induce activation of resting hepatic stellate cells (HSCs) into myofibroblast-like cells with the expression of α-smooth muscle actin filaments as a characteristic marker^11–14^. The activated stellate cells lose their lipid droplets (vitamin A), rapidly proliferate and dramatically upregulate a number of genes, especially for collagens, fibronectins, laminin, hyaluronic acid, and start increased synthesis of connective tissue proteins, markedly collagens^15–17^. This results in excessive deposition of several connective tissue proteins, mainly collagens in the hepatic parenchyma that leads to fibrosis. The uncontrolled and extensive fibrosis could produce distortion of normal architecture of the liver leading to nodular formation and cirrhosis. The repeated chronic injury and cellular regenerative events could result in genomic aberrations and mutations of oncogenes or tumor-suppressor genes leading to the development of hepatocellular carcinoma (HCC)^18–23^.

## *N*-Nitrosodimethylamine

**Fig. 1:**
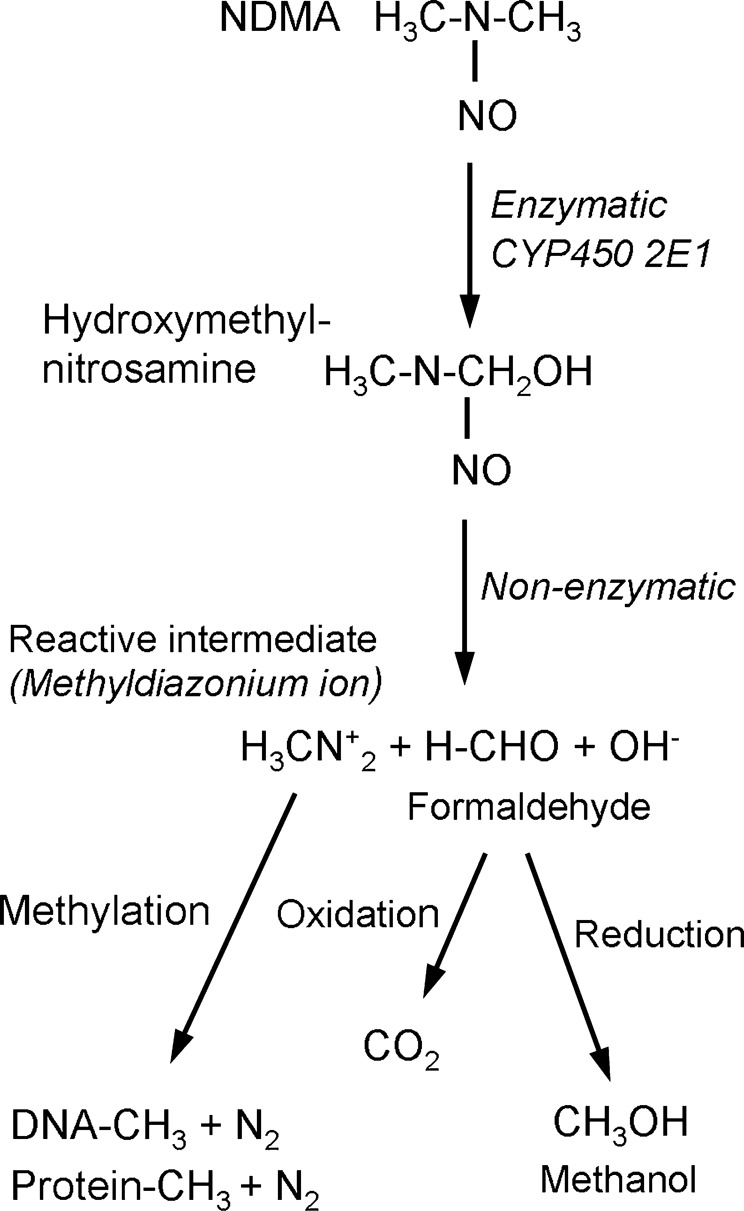
Metabolic activation and degradation of *N*-nitrosodimethylamine (NDMA) in liver. The metabolic degradation of NDMA produces formaldehyde and methanol, and the alkylating intermediate reacts with nucleic acids and proteins to form methylated macromolecules

*N*-Nitrosodimethylamine, (NDMA, (CH_3_)_2_N_2_O, Mol. wt. 74.08) also known as dimethylnitrosamine (DMN), is a byproduct of several industrial processes and is present in trace amounts in tobacco smoke condensates^24–26^. It is formed by the interaction of nitrate with dimethylamine and by the action of nitrite reducing bacteria^27,28^. Some of the physical, chemical, and biological properties of NDMA are presented in Table 1. Barnes and Magee first reported its hepatotoxicity following an industrial accident of liver cirrhosis^29^. NDMA is characterized as a potent hepatotoxin, carcinogen, and mutagen^4,30–32^. The toxicities produced by NDMA and related nitrosamines are mediated by reactive metabolic intermediates and not by the parent compound^32,33^. NDMA targets primarily the liver, which contains the necessary enzymes for its metabolic activation. Metabolism in the liver is by the microsomal membrane-bound enzyme, cytochrome P-450 2E1^34–36^. Its metabolic half-life is <10 min in rodents and about 20 min in non-human primates^37,38^. Activation and degradation of NDMA produces formaldehyde and methanol and an alkylating intermediate that reacts with nucleic acids and proteins to form methylated macromolecules (Fig. 1). It has been demonstrated that NDMA and other nitrosamines are metabolized in vitro by liver homogenates to yield the corresponding aldehyde and a chemically reactive alkylating species^39^. NDMA methylates proteins^40,41^ and DNA^42^ and forms specific DNA adducts^43,44^. In vitro preparations of human liver slices can also metabolize NDMA and methylates its DNA in the same order as rat liver slices^45^.

**Table 1:** Physical, chemical, and biological properties of *N*-Nitrosodimethylamine

Property	Value/description
Chemical Abstract Systems (CAS) Number^a^	62–75–9
Chemical formula	C_2_H_6_N_2_O
Physical description (physical state at room temperature)	Yellow liquid with no distinct odor
Molecular weight	74.083 g/mol
Solubility in water	290 g/L at 20 ^o^C
Specific gravity/density at 20 ^o^C/4 ^o^C	1.0048 g/mL
Melting point	<25 °C (estimated)
Boiling point	153.1 ^o^C (307.5 °F)
Vapor pressure at 20 ^o^C	2.7 mm Hg
Flash point	61.0 ^o^C (141.8 °F)
LD_**50**_ (rat)	37.0 mg/kg (oral)
Biohazards	Highly hepatotoxic, carcinogenic

*g/mol* grams per mole, *g/mL* grams per milliliter, *°C* degrees Celsius,

*°F* degrees Fahrenheit, *mm Hg* millimeters of mercury, LD_**50**_ Median lethal dose (lethal dose 50%), *mg/kg* milligram per kilogram

^a^Source

## *N*-Nitrosodimethylamine-induced hepatic fibrosis and liver cirrhosis

The NDMA-induced canine model of hepatic fibrosis was first developed by Madden et al^46^. Later it was demonstrated that serial administrations of NDMA in rats could produce a reproducible model of hepatic fibrosis, cirrhosis, and portal hypertension, as seen in human beings^47^. Afterwards Jezequel and coworkers conducted a pioneering study on various aspects of NDMA-induced hepatic fibrosis with special emphasis to pathophysiology and immunohistochemistry and demonstrated that it is a good and reproducible animal model, and appropriate for the study of the early events associated with the development of hepatic fibrosis^48–52^. Furthermore, the model has been employed recently to investigate various aspects of the molecular pathogenesis of hepatic fibrosis and to study therapeutic approaches including the arrest of activation of stellate cells^53–60^. Over the last 20 years, we have extensively studied various biochemical and pathophysiological aspects of the pathogenesis of NDMA-induced hepatic fibrosis in rats and mice involving glycoprotein metabolism^17^, collagen biosynthesis and metabolism^4–6^, LDH isoenzymes^61^, biochemical abnormalities^62^, oxidative stress and osteopontin^10,63–65^, hyaluronic acid and hyaluronidase^66,67^, mineral and trace element metabolism^68–70^, antioxidants^10,71,72^ and gene therapy^13,73,74^, lysosomal fragility^75,76^, and the role of metalloproteinases^14,77,78^. These studies demonstrated that NDMA-induced model of hepatic fibrosis and early cirrhosis in rats is an easy and quick model to study the molecular mechanisms involved in the pathogenesis of liver fibrosis and cirrhosis of human beings.

## Events involved in the pathogenesis of NDMA induced hepatic fibrosis and cirrhosis

**Fig. 2:**
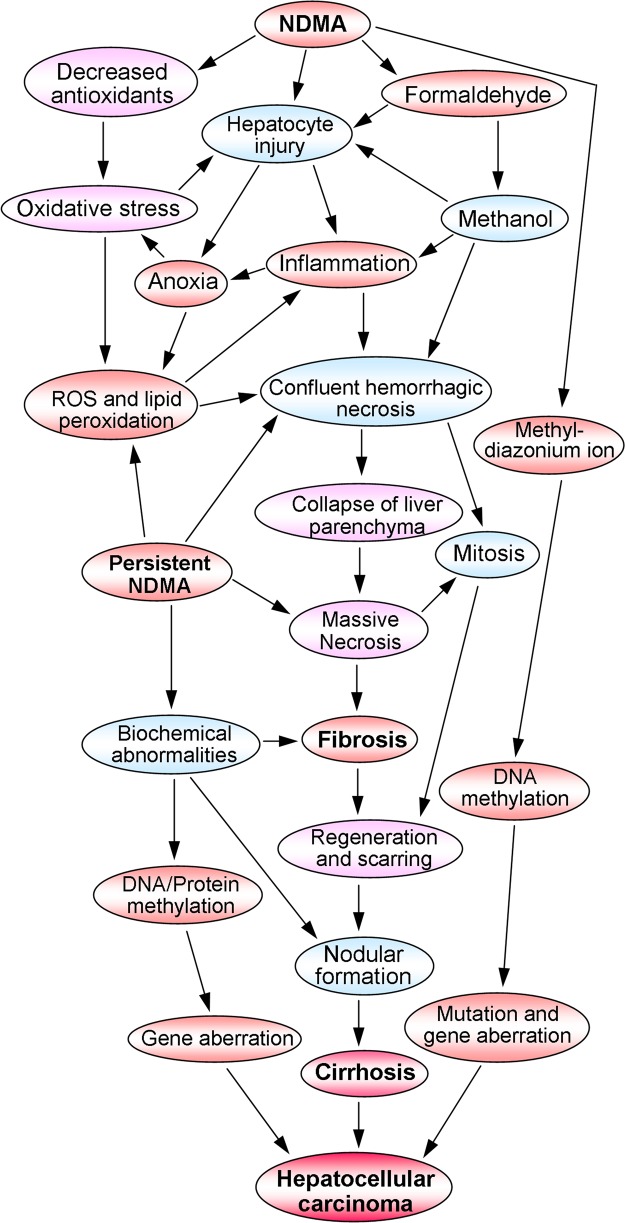
Schematic representation of the sequence of events involved in the pathogenesis of N-nitrosodimethylamine (NDMA) induced hepatic fibrosis, cirrhosis, and hepatocellular carcinoma. The metabolic activation and detoxification of NDMA cause hepatocyte injury, inflammation, neutrophilic infiltration, and massive hepatic necrosis, which results in oxidative stress and production of reactive oxygen species. These processes induce activation of hepatic stellate cells and increased synthesis of connective tissue components, especially collagens that end up in hepatic fibrosis. The chronic liver injury and perpetual fibrosis lead to liver cirrhosis, which could develop into hepatocellular carcinoma

A schematic representation of the sequence of events involved in the pathogenesis of NDMA-induced hepatic fibrosis, liver cirrhosis, and the ultimate hepatocellular carcinoma is presented in Fig. 2. The metabolic activation and detoxification process of NDMA induces liver injury in multiple ways. The enzymatic degradation of NDMA produces hydroxymethylnitrosamine, which in turn non-enzymatically converted into formaldehyde and methanol (Fig. 1). Both compounds are highly toxic to the liver and initiates severe inflammation and confluent hemorrhagic necrosis. These processes results in extreme oxidative stress and production of reactive oxygen species (ROS) that further contributes to hepatocyte damage and necrosis. Furthermore, NDMA decreases catalase and glutathione peroxidase, the major antioxidant enzymes present in the liver^79–81^. We have observed that NDMA treatment dramatically decreases serum and liver concentrations of ascorbic acid, another major antioxidant^10^. In addition, the metabolic generation of reactive intermediate, the methyl carbonium ion by NDMA damages hepatic tissue in multiple ways and triggers fibrogenesis.

The persistent treatment of NDMA further increases oxidative stress and lipid peroxidation that enhances hemorrhagic necrosis and collapse of liver parenchyma. The extensive panlobular and multilobular necrosis lead to massive hepatic necrosis, which in turn initiates mitosis and hepatic regeneration. On the other hand, the resting HSCs transform into myofibroblast like cells and start extensive synthesis of connective tissue proteins. This causes deposition of mature collagen fibrils in the extracellular matrix of the liver and results in hepatic fibrosis. All these processes lead to condensation of hepatic reticulin framework, production of granulation tissue, and ultimately scar formation^82–84^. The ischemic consequences of the hepatic tissue and confluent necrosis amplify the process of nodular regeneration and drive towards to liver cirrhosis. The repeated tissue repair and regeneration process can lead to aberrations and mutations in genes and end up in development of HCC^85–88^. Alternatively, the methyl carbonium ions produced during metabolic degradation of NDMA methylate the hepatocyte DNA that results in gene mutation and trigger HCC^89–91^.

## Cellular interactions and molecular mechanisms in the pathogenesis of NDMA-induced hepatic fibrosis

**Fig. 3:**
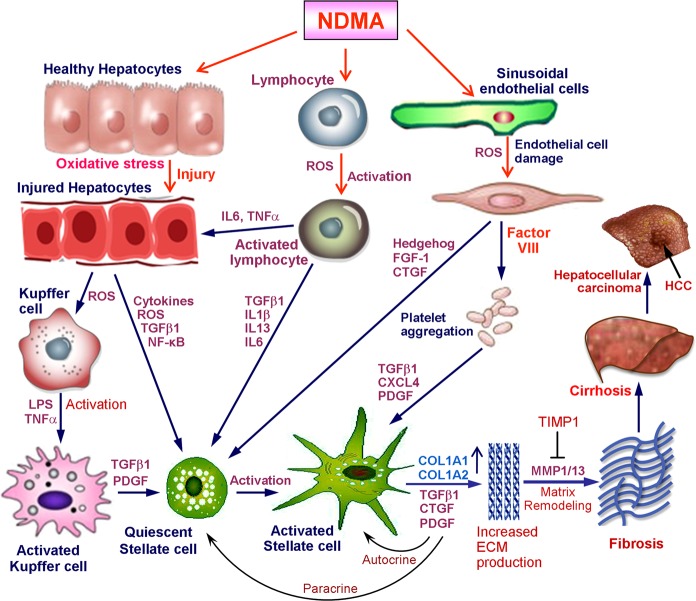
Schematic representation of the cellular interactions and molecular mechanisms involved in the pathogenesis of *N*-nitrosodimethylamine induced hepatic fibrosis. Hepatocyte injury leads to inflammation and generation of reactive oxygen species that in turn activate Kupffer cells. The activated Kupffer cells produce the most potent fibrogenic factor TGF-β1, which activate the quiescent hepatic stellate cells into myofibroblast like cells with the expression of α-smooth muscle actin filaments. Alternatively, metabolism of NDMA cause activation of lymphocytes and injury to sinusoidal endothelial cells which produce potent fibrogenic factors like TGF-β1, CTGF, and FGF-1 and cytokines and growth factors such as NF-κB, IL-1β, IL-6, IL-13, IL-22, and CXCL4 which altogether contribute the activation of resting hepatic stellate cells. The activated and transformed stellate cells express and upregulate hundreds of genes, especially for collagens and other connective tissue proteins. The excessive synthesis and deposition of these proteins, specifically fibril forming collagens in the extracellular matrix of the liver leads to fibrosis and cirrhosis and ultimately to HCC

A schematic representation of the cellular interactions and molecular mechanisms involved in the pathogenesis of NDMA-induced hepatic fibrosis is presented in Fig. 3. An injury to the liver produces a response from various types of cells and alters cell–cell and cell–matrix interactions. Such a response leads to inflammation accompanied by the infiltration of lymphocytes, monocytes, granulocytes, and macrophages into the space of Disse^92–94^. Treatment with NDMA injures both parenchymal and non-parenchymal cells in multiple ways, produces inflammation, and generates oxidative stress and reactive oxygen species (ROS). In addition, NDMA could produce increased gut permeability, which in turn accelerates the entry of bacterial endotoxin (lipopolysaccharide) into the blood stream, which interacts with Kupffer cells in the liver and activates them^95,96^. Lipopolysaccharide (LPS), a component of the cell walls of some gram-negative bacteria that normally inhabit the intestine, is one substance that can effectively activate Kupffer cells^97,98^. The activated Kupffer cells produce several cytokines and growth factors such as tumor necrosis factor-α (TNF-α), transforming growth factor-β1 (TGF-β1), platelet derived growth factor (PDGF), and interleukin (IL)−1β which in turn activate and transform the quiescent HSCs into myofibroblast like cells^99–101^.

Ingestion or administration of NDMA triggers the immune system and activate the lymphocytes which in turn produce various pro-inflammatory cytokines such as IL-1β, IL-6, IL-22, interferon-γ (IFN-γ), and TNF-α^102–104^. The pro-inflammatory cytokines trigger hepatocytes to activate downstream signaling pathways such as nuclear factor-kB (NF-kB) and TGF-β, which in turn induce activation of resting hepatic stellate cells. On the other hand, the activated lymphocytes produce a variety of cytokines and growth factors including TGF-β1, IL-1β, IL-6, and IL-13, which directly activate and transform the quiescent stellate cells^105–107^. Alternatively, the sinusoidal endothelial cell damage caused by ROS and other methods during NDMA treatment produce potent fibrogenic factors such as connective tissues growth factor (CTGF) and fibroblast growth factor-1 (FGF-1) and may also induce hedgehog signaling^108–110^. Both CTGF and FGF-1 induce activation of stellate cells into myofibroblast like cells. In addition, it was shown that hedgehog signaling in liver sinusoidal endothelial cells regulate capillarisation during fibrogenesis^110,111^. Damage to the endothelial cells could release several factors including Factor VIII that cause platelet aggregation and further produce TGF-β1 and platelet derived growth factor (PDGF) and CXC chemokine ligand 4 (CXCL4)^112,113^. Furthermore, cytosolic fragments released by injured hepatocytes could directly stimulate perisinusoidal cells and induce production several fibrogenic and growth factors that in turn transform the resting stellate cells into myofibroblast like cells^112,114^.

The activation and transformation of quiescent HSCs into large myofibroblast like cells with the loss of fat globules and expression of alpha smooth muscle actin (α-SMA) is a very crucial and important step in hepatic fibrogenesis^115,116^. Increased level of intracellular cAMP is required for the conversion of HSCs into myofibroblasts and their proliferation^117,118^. The transformed HSCs further produce several cytokines and growth factors, especially TGF-β1, CTGF, and PDGF which in turn further stimulate more production of cytokines and growth factors through the autocrine mechanism and also transforms the remaining quiescent stellate cells by the paracrine mechanism (Fig. 3). The process of activation of quiescent HSCs is accompanied with the expression and upregulation of 100 s of genes, especially for collagens and other extracellular matrix proteins^11,119,120^. The net result is excessive and non-regulated synthesis and deposition of connective tissue components especially fibril forming collagens in the extracellular space of the liver^4,121,122^. This coincides with the development of basement membrane formed with type IV collagen and laminin in the space of Disse and decreases the number of fenestrations of sinusoidal endothelial cells^123^. The defenestration of endothelial cells leads to increased diffusional barrier and interferes with the transport of nutrients to hepatocytes contributing to the development of portal hypertension and impairment of liver functions.

Matrix metalloproteinases (MMPs) are capable of degrading extracellular matrix proteins including all forms of native collagens and play a prominent role in remodeling of connective tissue matrix during pathogenesis of hepatic fibrosis^14,124,125^. The major interstitial collagenases that degrade native fibrillar collagens in human are MMP-1 and MMP-13. However, mice and rats do not possess a homologous to human MMP-1 gene^126^. We have demonstrated that NDMA-induced fibrotic fiver collagen is more cross-linked than normal liver collagen and the deposition of type III collagen is more prominent than type I collagen^6^. The extreme necrosis of hepatic parenchyma during persistent NDMA administration leads to decreased enzyme synthesis and thus reduced interstitial collagenases levels. On the other hand, tissue inhibitor of metalloproteinases (TIMPS), especially TIMP-1 is markedly upregulated during pathogenesis of hepatic fibrosis, which inhibits the activity of MMPs^78,127–129^. Thus there is impairment in the balance between synthesis and degradation of collagens in multiple ways and the net result is deposition of excessive amount of fibril forming collagens in the extracellular matrix of the liver. Once mature collagen fibrils are deposited in the extracellular compartment of the liver, it will form stable inter and intra cross-linking and lead to hepatic fibrosis^130^. The persistent stimulus results in repeated confluent necrosis, wound healing, scarring, and nodular formation, which could lead to liver cirrhosis and ultimate death.

## Summary and conclusion

Hepatic fibrosis is the result of excessive synthesis and deposition of connective tissue proteins, especially fibril forming collagens (collagens type I and type III) in the extracellular matrix of the liver. An abnormal wound healing process in response to a chronic liver injury is responsible for the pathogenesis of hepatic fibrosis and subsequent liver cirrhosis leading to hepatocellular carcinoma and ultimate death. The repeated stimuli involved in the initiation of fibrosis lead to oxidative stress and generation of reactive oxygen species along with marked decrease of antioxidant status. These processes results in cellular injury and initiate inflammatory responses releasing a variety of cytokines and growth factors that serve as mediators of molecular events involved in the initiation of fibrosis. The key event involved in the pathogenesis of hepatic fibrosis is the activation and transformation of resting hepatic stellate cells into myofibroblast like cells and subsequent upregulation of numerous genes, especially genes for connective tissue proteins. The transformed stellate cells abandon its vitamin-A storage function and start excessive synthesis and deposition of connective tissue components especially collagens, glyocoproteins, and glycosaminoglycans in the extracellular matrix of the liver. Extensive and uncontrolled fibrosis results in distortion of lobular architecture of the liver leading to nodular formation and cirrhosis. The chronic injury and regeneration process could results in genomic aberrations and mutations that lead to the development of hepatocellular carcinoma. Unraveling the precise molecular mechanism involved in the pathogenesis and progression of hepatic fibrosis would help to design successful therapeutic approaches that could prevent liver cirrhosis and subsequent hepatocellular carcinoma.
